# Diel Activity Patterns of Bobcats and Domestic Cats in the Houston Metropolitan Area

**DOI:** 10.17912/micropub.biology.001911

**Published:** 2026-06-13

**Authors:** Leila Alavi Naini, Natalie Linde, Sarah Malik, Yasin Syed, Ann Oliver Cheek

**Affiliations:** 1 Honors College, University of Houston, Houston, TX, United States; 2 Department of Biology and Biochemistry, University of Houston, Houston, TX, United States; 3 Department of Biomedical Engineering, University of Houston, Houston, TX, United States

## Abstract

We investigated whether bobcats ( *Lynx rufus)* and domestic cats ( *Felis catus* ) exhibit distinct daily activity patterns or use different habitats in the Houston, Texas metropolitan area. Motion-activated cameras were deployed at 33 sites for 16 one-month sampling periods from 2020 - 2024. Bobcats exhibited primarily nocturnal activity wherever they were present. Domestic cats were primarily nocturnal at sites where no bobcats were detected. Bobcats and domestic cats overlapped at sites with a mixture of forest and developed land and domestic cats shifted to more daytime activity. Both temporal and spatial niche partitioning appear to facilitate predator coexistence in urban landscapes.

**Figure 1. Spatial and diel distribution of bobcats and domestic cats:** (A) US state of Texas. Red square indicates the city of Houston. (B) Map of 33 camera sites in the Houston, TX metropolitan area where at least one bobcat or domestic cat was detected during the four-year period, October 1, 2020 to August 31, 2024. Blue markers indicate sites where only bobcats were detected, pink markers indicate sites where only domestic cats were detected, and white markers represent locations where both species were detected. (C and D) Diel activity of bobcats (blue) and domestic cats (pink) across a 24h period at sites where only one of the species occurred (C) and at sites where both species occurred (D). Bar heights in panels C and D are the proportion of species-specific observations per hour.

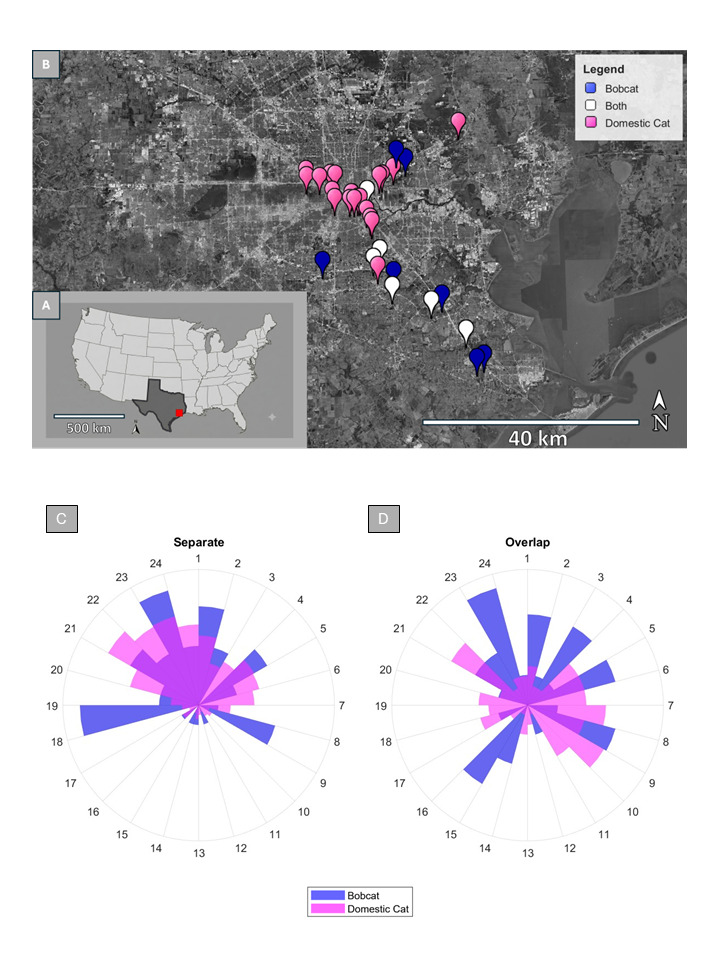



## Description

Predators may reduce interference competition by avoiding interaction with each other, either through spatial or temporal separation. Where spatial overlap occurs, they may change their active period and their preferred prey (Kronfeld-Schor and Dayan, 2003). Human land use also influences predator interactions because larger predators tend to move away from human development or shift to primarily nocturnal activity (Gaynor et al., 2018). 

Bobcats ( *Lynx rufus* ) and free-ranging, unowned domestic cats ( *Felis catus* ) are carnivores with similar diets consisting of a high proportion of rodents and rabbits (Hass, 2009; Kitts-Morgan, 2015; Loss et al., 2013). Habitat preferences create partial spatial separation of these two predators. Bobcats prefer areas with less human development (Horne et al., 2009), while domestic cats are more likely to occur in areas with higher human population density (Herrera et al., 2022). Even in rural areas, domestic cats tend to remain near human infrastructure (Dunford et al., 2024). Bobcats and domestic cats co-occur in rural, suburban, and exurban landscapes (MacDougall and Sander, 2022; Ordenana et al., 2010; Wait et al., 2018), raising the possibility that temporal separation could reduce interference competition.

Bobcats are active during the day and at night, with more detections at night in exurban areas (Mayer et al., 2023; Poisson et al., 2023). Free-ranging domestic cats are active throughout the day and night in some locations (Dunford et al., 2024; Germain et al., 2008; Herrera et al., 2022), but are primarily nocturnal in others (Krauze-Gryz et al., 2012). Although several studies of urban mammal communities mention detections of both domestic cats and bobcats in the same region, no studies have compared space use or activity periods between these two felids. 

The objective of this study was to compare diel activity patterns of bobcats and domestic cats where each species occurred without the other and where they occurred together across a large urban area. Motion-activated trail cameras were deployed in parks, cemeteries, golf courses, and natural areas along two transect lines in the Houston, TX metropolitan area (Fig 1A and B). The Houston metropolitan area covers 22,891 km ^2^ with a population of more than 7 million people (Census Reporter, 2025). To sample activity throughout the year, camera traps were deployed in January (winter), April (spring), July (summer), and October (fall) (Magle et al., 2019). Detection data were pooled across 16 sampling seasons (2020 to 2024) to obtain a general pattern of diel activity for each species.

During 14,386 trap-days from October 1, 2020 to August 31, 2024, we collected a total of 81 bobcat detections and 795 domestic cat detections across 33 sites. Bobcats alone were detected at seven sites, all of which were more than 13.7 km outside of downtown Houston (Fig 1B). Across these seven sites, developed land accounted for a mean of 50% (± 19%, 1 s.d.) of the land cover within a 1 km radius of the camera and forest accounted for 27% (± 13%) of land cover. Domestic cats alone were detected at 19 sites. Nearly all of these sites were within 12 km of downtown Houston, inside the innermost multi-lane beltway, interstate 610 (Fig 1B). Across these 19 sites, developed land constituted 82 ± 14% (mean ± 1 s.d.) of land cover within the camera buffer, whereas forest accounted for 12 ± 13%). Bobcats and domestic cats overlapped at seven sites where 65% (± 16%) of land cover was developed land and 16% (± 7%) was forest ( Fig. 1B ). Spatial overlap occurred at sites located 6 – 44 km outside of downtown Houston.

The diel activity patterns of bobcats and domestic cats were similar where only one species occurred without the other (Fig 1C, n = 49 bobcat detections; n = 696 domestic cat detections; Watson’s U2 = 0.0753, p < 0.20). Both species were more active at night than during the day, with high overlap between their diel activity patterns, overlap coefficient Δ̂ = 0.852. At sites occupied only by bobcats, 67% of bobcat detections occurred at night. At sites with domestic cats but no bobcats, 85% of domestic cat detections occurred at night. Where the two species co-occurred spatially, bobcats were still primarily active at night. In contrast, only 51% of domestic cat detections occurred at night.  Activity patterns of the two species overlapped much less where they co-occurred, Δ̂ = 0.647. Domestic cat diel activity differed significantly from bobcat activity (Fig 1D, n = 32 bobcat detections; n = 99 domestic cat detections; Watson’s U2 = 0.189, p < 0.05).

Bobcats and domestic cats had a similar primarily nocturnal activity pattern at sites where only one felid occurred. Both species prey on small nocturnal mammals, including rodents and rabbits. Nocturnal activity may be a strategy that simultaneously matches peak prey activity (Kronfeld-Schor and Dayan 2003) and avoids peak human activity (Gaynor et al., 2018). Where domestic cats and bobcats occupied the same sites, domestic cats shifted their diel activity toward pre-dawn through early morning (03:00 – 10:00) and late day through early night (16:00 – 21:00) with very little activity in the middle of the day or night. This shift is evident in the smaller Δ̂ overlap coefficient for domestic cat and bobcat activity at sites where both species were present. In other regions where larger and smaller felids overlap spatially, the smaller species tends to shift or even restrict its active period relative to the larger species to avoid being eaten or to exploit different prey (Hass, 2009). Shifting to more activity in low light periods at dawn and dusk may allow free-ranging domestic cats to reduce potential encounters with bobcats, thereby reducing the threat of predation. Additionally, changing their active period may allow domestic cats to exploit prey with a similar active period, particularly ground-feeding or ground-nesting birds. Across the United States, birds make up approximately 10% of prey killed by free-ranging domestic cats (Loss et al., 2013). Domestic cats are often assumed to depend on humans for food, but few domestic cats observed in this study were collared, raising the possibility that they survive by hunting. 

A combination of spatial and temporal partitioning appears to facilitate coexistence of bobcats and free-ranging domestic cats in the Houston metropolitan area. Bobcats were detected more frequently in areas with ≤ 65% developed land cover, while domestic cats were detected more frequently in areas with ≥ 65% developed land cover. Where domestic cats and bobcats overlapped spatially, domestic cat activity shifted from primarily nocturnal to the night-day boundary. Temporal niche partitioning may allow domestic cats to avoid interspecific competition with sympatric bobcats. 

## Methods

Trail cameras (Bushnell Trophy Cam HD model 11874C, Bushnell Core Low Glow model 119936C, Stealth Cam G45NG Pro) were mounted at approximately 1 meter above ground to optimize detection of mammals larger than 30 g. Camera sites were spaced at least 1 km apart to minimize detecting the same individual repeatedly. Cameras operated for a minimum of 30 consecutive days per sampling period four times per year - January, April, July, and October. 

All photos were annotated independently by two volunteers, with identification discrepancies resolved by an expert reviewer. Filename, date, time, and location were recorded for each detection. Detections from 16 sampling seasons (10/1/2020 - 8/31/2024) were analyzed. Analyses were conducted in R (R Core Team, 2026). Data management was performed using the readr (Wickham et al., 2026) and dplyr (Wickham et al., 2026) packages. Timestamps in the raw data were recorded in Universal Coordinated Time (UTC). UTC timestamps were converted to local time using the lubridate package (Grolemund & Wickham, 2011), transformed to radians, and converted to solar time using the sunTime function in the overlap package (Meredith et al., 2024). SunTime standardizes observation times relative to sunrise and sunset based on observation date and geographic coordinates. Temporal overlap between bobcat and domestic cat activity was estimated as Δ̂ using the overlap package. Overlap between species-specific activity patterns was calculated separately for sites where each species occurred alone versus sites where both species occurred. Watson’s U2 test was used to compare diel activity between bobcats and domestic cats at sites occupied by only one of the species and at sites occupied by both species. Occupancy was defined as at least one confirmed detection occurring at a site during the study period (Oct 2020 – Aug 2024). Watson's U2 test was performed in MATLAB 24.1.0.2537033 (2024b). 

Camera locations were mapped and land cover within a 1 km radius around each camera was visualized using Google Earth Pro on Desktop (https://www.google.com/earth/about/versions/#earth-pro). Land cover classes surrounding each site were characterized according to the modified Anderson Level II classification system (USGS, 2024). Area occupied by each land cover type was quantified using the freehand polygon measurement tool in Google Earth Pro on Desktop. Downtown Houston was defined as the location of the 1910 Harris County Courthouse, 29.760890, -95.359704. Map inset showing Texas within the United States was created using Gemini Flash 3.5 and specifying datum WGS 84. 

The Institutional Animal Care and Use Committee reviewed this study and determined that no protocol was required because animals are not handled nor are they disturbed by the cameras.

Animal detection data, MatLab script and R script are available at datadryad.org, DOI: 10.5061/dryad.s4mw6m9jg
